# Second-Generation Antipsychotics and Extrapyramidal Adverse Effects

**DOI:** 10.1155/2014/656370

**Published:** 2014-06-03

**Authors:** Nevena Divac, Milica Prostran, Igor Jakovcevski, Natasa Cerovac

**Affiliations:** ^1^Institute of Pharmacology, Clinical Pharmacology and Toxicology, Faculty of Medicine, University of Belgrade, Dr. Subotica 1, 11000 Belgrade, Serbia; ^2^University Medical Center Hamburg-Eppendorf, Center for Molecular Neurobiology, Falkenried 94, 20251 Hamburg, Germany; ^3^Clinic for Neurology and Psychiatry for Children and Youth, Faculty of Medicine, University of Belgrade, Dr. Subotica 6a, 11000 Belgrade, Serbia

## Abstract

Antipsychotic-induced extrapyramidal adverse effects are well recognized in the context of first-generation antipsychotic drugs. However, the introduction of second-generation antipsychotics, with atypical mechanism of action, especially lower dopamine receptors affinity, was met with great expectations among clinicians regarding their potentially lower propensity to cause extrapyramidal syndrome. This review gives a brief summary of the recent literature relevant to second-generation antipsychotics and extrapyramidal syndrome. Numerous studies have examined the incidence and severity of extrapyramidal syndrome with first- and second-generation antipsychotics. The majority of these studies clearly indicate that extrapyramidal syndrome does occur with second-generation agents, though in lower rates in comparison with first generation. Risk factors are the choice of a particular second-generation agent (with clozapine carrying the lowest risk and risperidone the highest), high doses, history of previous extrapyramidal symptoms, and comorbidity. Also, in comparative studies, the choice of a first-generation comparator significantly influences the results. Extrapyramidal syndrome remains clinically important even in the era of second-generation antipsychotics. The incidence and severity of extrapyramidal syndrome differ amongst these antipsychotics, but the fact is that these drugs have not lived up to the expectation regarding their tolerability.

## 1. Background

Antipsychotic drugs are the cornerstone of the pharmacological treatment of schizophrenia. The introduction of the first antipsychotic chlorpromazine in 1952 marked the new era in psychopharmacology [1]. However, those early antipsychotics, now referred to as first-generation antipsychotics (FGAs), such as chlorpromazine, haloperidol, or fluphenazine, though effective in relieving positive symptoms of the disease, have some serious limitations. Lack of efficacy regarding negative symptoms and the adverse effects, especially extrapyramidal symptoms (EPS), are serious drawbacks of these drugs. The development of newer antipsychotics (risperidone, olanzapine, quetiapine, etc.) since 1990s was met with great expectations. These novel antipsychotics, now referred to as second-generation antipsychotics (SGAs), have been modeled on the prototype drug clozapine [2].

Clozapine was the first antipsychotic that proved to be efficacious in treatment-refractory schizophrenia [3], but it was also the first antipsychotic devoid of EPS. However, the ability of clozapine to cause agranulocytosis as a serious adverse effect led to voluntary withdrawal of the drug by the manufacturer, with subsequent reintroduction in 1989, followed by strict regulation regarding indications and white blood cells count followup [4]. The efficacy of clozapine and its inability to produce EPS were motives for the development of similar antipsychotics, but with the safer profile. Second-generation antipsychotics such as olanzapine, risperidone, quetiapine, and more recently ziprasidone and aripiprazole soon became the mainstay of the treatment of schizophrenia, despite their higher costs and inconsistency of the data showing their superior efficacy versus FGAs [5, 6].

Clozapine, as the first SGA, actually discredited the theory that EPS are an unavoidable accompaniment of antipsychotic efficacy. Previously, EPS were considered as an essential component of antipsychotic “neuroleptic” effect. The association of antidopaminergic (D2) potency, antipsychotic effect, and EPS (due to loss of dopamine in the extrapyramidal part of the central nervous system) was the foundation for the dopamine hypothesis of schizophrenia [7, 8]. The ability of a substance to induce EPS experimentally was considered as proof of its antipsychotic potential. However, dopamine hypothesis of schizophrenia became obsolete with the introduction of clozapine and other SGAs.

All antipsychotic agents have some degree of antagonistic affinity for dopaminergic D2 receptors. It was shown that first-generation antipsychotics, though known to block other receptors, not only exert their antipsychotic, but also their extrapyramidal effects, primarily by binding to D2 receptors in the central nervous system. First-generation antipsychotics produce their therapeutic (antipsychotic) effect at 60–80% of D2 occupancy, while the 75–80% of D2 receptor occupancy leads to the acute EPS [9–11]. Therefore, the overlap between desired and adverse D2 receptor occupancy is mostly unavoidable with FGAs. On the other hand, the therapeutic effects of SGAs are attributable also to some degree to D2 antagonism, but more to blockade of certain serotonin (mostly 5HT2A) receptors. Surprisingly, clozapine, as the most effective antipsychotic so far, has the lowest D2 affinity (Table 1). It was also suggested and shown in animal models that SGAs actually bind to and dissociate from D2 receptors in an atypical manner (Kapur, 2001). Loose binding to and fast dissociation of SGAs from D2 receptors may be the cause of their lower EPS propensity [12]. The affinity of antipsychotic drugs for D2 receptors is shown in Table 1. While the antipsychotic effect of FGAs correlates with D2 affinity, that is not the case with SGAs.

The efficacy of a pharmacological treatment cannot be interpreted independently from its adverse effects profile. Better tolerability of SGAs was considered as one of their major advantages as a class [7]. The idea of treating schizophrenia without producing EPS was very attractive for psychiatric care professionals, as well as for the patients. However, post-clozapine SGAs have not fully lived up to these expectations and intolerability due to the fact that EPS remain a considerable problem in the treatment of schizophrenia [7, 13]. It is now evident that all SGAs, apart from clozapine, have propensity to cause certain degree of EPS. The results of recent clinical trials and meta-analyses have shown that there is no advantage of SGAs regarding tolerability and effectiveness compared with FGAs [13, 14]. Also, postmarketing followup of SGAs surfaced other adverse effects such as weight gain and metabolic side effects. However, notable metabolic side effects are also caused by FGAs and the higher cardiometabolic risk of SGAs versus FGAs has not been confirmed [15]. Therefore, the oversimplified distinction of antipsychotic drugs classes, in which FGAs are responsible for EPS and SGAs for metabolic side effects, though ingrained in clinical practice, is actually not supported by recent findings [1, 16].

This review summarizes the recent reported results regarding the risk of EPS development in patients treated with different classes of antipsychotic drugs.

## 2. Extrapyramidal Symptoms

EPS include acute dystonias, akathisia, Parkinsonism, and tardive dyskinesia (TD). EPS are serious, sometimes debilitating and stigmatizing adverse effects, and require additional pharmacotherapy. EPS develop into two phases. Early, acute EPS most often develop upon the beginning of treatment with antipsychotics or when the dose is increased. The later-onset EPS usually occur after prolonged treatment and present as tardive dyskinesia (TD). The motor manifestations include akathisia (restlessness and pacing), acute dystonia (sustained abnormal postures and muscle spasms, especially of the head or neck), and Parkinsonism (tremor, skeletal muscle rigidity, and/or bradykinesia) [13, 17]. TD is characterized by involuntary, repetitive facial movements such as grimacing, tongue protruding, oculogyric crisis, and lips puckering, as well as torso and limb movements. Acute EPS are one of the main causes of poor adherence to antipsychotic treatment due to the reversibility of symptoms, while late-onset TD has the most serious impact on patients and caregivers with respect to quality of life [18, 19]. TD may persist after the discontinuation of treatment or even be irreversible. It is estimated that approximately 50% of patients treated with high-potency FGAs (such as haloperidol) develop acute EPS within the first several days of treatment. The prevalence of TD is somewhat less known due to differences in design and methodologies among studies that have investigated this problem [13, 20, 21]. Prevalence of TD has been reported to be 0.5% to 70% of patients receiving FGAs, with the average rate being between 24% and 30% [22, 23].

Acute EPS usually respond to dose reduction of the antipsychotic agent or require additional pharmacological treatment.

Acute dystonia occurs within first few days after the initiation of the antipsychotic treatment and can be effectively prevented or reversed with anticholinergic drugs such as biperiden [24–26]. Risk factors for acute dystonia are young age and male gender, history of substance abuse, and family history of dystonia [27, 28]. Acute dystonia is common with FGAs such as haloperidol [29] and less common with SGAs. It is reported that approximately 7.2% treated with long-acting parenteral risperidone develop acute dystonic reactions [30]. Also, case reports regarding acute dystonia after initiation of antipsychotic treatment with aripiprazole and ziprasidone have been published [31, 32].

Akathisia is very common (about one half of all cases of EPS), poorly understood, and difficult to treat. It occurs mostly within the first three months of treatment. Akathisia does not respond to anticholinergic medication, but antipsychotic dose reduction, liposoluble beta adrenergic blockers, and benzodiazepines have proved effective [24, 25]. The rough estimation is that about 25% of patients treated with FGAs develop akathisia, but it is also common with SGAs. Some researchers suggest that akathisia rates do not differ between FGAs and SGAs [24]. It was previously suggested that SGAs clozapine and quetiapine carry the lowest risk for akathisia, yet it was not confirmed in some blinded reviews [33]. Also, the Clinical Antipsychotic Trials of Intervention Effectiveness (CATIE) study as a randomized, partially open-label study in which efficacy and side effects of multiple SGAs with an FGA perphenazine showed that akathisia remains a problem with SGAs, though at lower rates compared to FGAs [24, 34]. Based on CATIE study, it appeared that risperidone and perphenazine, for example, both cause akathisia in 7% of patients. Further analysis of the CATIE study data revealed no difference between any of the antipsychotics tested in this study regarding incidence of akathisia and other EPS in patients with chronic schizophrenia during maintenance of antipsychotic treatment for up to 18 months [35]. However, the well-known limitations of the CATIE (the choice of an intermediate-potency FGA perphenazine, the nonrandomized allocation of patients with the tardive dyskinesia to a SGA treatment) should be considered when interpreting these results.

Parkinsonism induced by antipsychotics occurs between few days and up to several months after the initiation of the treatment. Risk factors for this type of Parkinsonism are age (elderly), gender (females), cognitive deficit, and early onset EPS [36]. Antipsychotic-induced Parkinsonism is considered a reversible condition although its duration is variable. The treatment of choice is not established, but dose reduction and anticholinergic drugs may be useful. However, anticholinergics should be avoided in the elderly patients due to their side effects such as cognitive deterioration, urinary retention, dry mouth, and risk of glaucoma exacerbation. Although switching to SGAs is often recommended in cases of Parkinsonism, the rates of Parkinsonism induced by SGAs (e.g., 26% with olanzapine) are lower than those with the FGAs (55% with haloperidol), but not negligible [37]. Other evidence shows virtually no advantages of SGAs compared to FGAs in relation to Parkinsonism as an adverse effect, especially when the potency and dose are considered. It was shown that high doses of SGAs (such as olanzapine, risperidone, or quetiapine) caused Parkinsonism in high doses at a similar rate as low-potency FGA (chlorpromazine), but the risk was 50% higher in high-potency FGA group [38]. In CATIE study, the results regarding Parkinsonism were also conflicting. CATIE study includes patients with previous tardive dyskinesia, who at baseline were excluded from perphenazine branch. This could lead to potential bias, meaning that patients with previous vulnerability to EPS were allocated exclusively to SGA branch. In order to avoid this potential bias, only patients without previous TD were included in comparisons for Parkinsonism. The proportion of patients showing no evidence of Parkinsonism at baseline who met at least one of the three criteria for Parkinsonism during the subsequent follow-up period revealed no substantial differences between treatment groups. At the 12-month followup, covariate-adjusted rates of Parkinsonism were 37%–44% for SGAs and 37% for perphenazine [35]. However, the choice of an intermediate-potency FGA (perphenazine) as a comparator in modest doses in CATIE could probably be responsible for the lack of significant difference between FGAs and SGAs regarding incidence of Parkinsonism. The Cost Utility of the Latest Antipsychotics in Schizophrenia Study Band 1 (CUtLASS-1) as a randomized controlled trial (RCT) that tested the hypothesis that the clinical and cost-effectiveness of SGAs is superior in individuals whose antipsychotic treatment is changed due to insufficient efficacy or side-effects of previous treatment. This study also did not show statistically significant difference between the treatment groups in terms of Parkinsonism between SGA and FGA patients [39] between SGA and FGA patients. The results were similar regarding akathisia. As in CATIE study, the main limitation of this study is the choice of FGA comparator. Haloperidol as the high-potency FGA was a rare choice at baseline, while sulpiride was the most common. Sulpiride is considered as an FGA with atypical properties and its low propensity for EPS is well established [40].

Tardive dyskinesia occurs after months or years of antipsychotic therapy. The risk of TD development is highest in the first five years of treatment with FGAs [24]. Leading risk factors for TD are increased age, non-Caucasian race, female gender, a history of diabetes, organic brain damage, and the presence of negative symptoms of schizophrenia [41]. TD can also occur spontaneously in patients diagnosed with schizophrenia at the rate of 0.5% per year [42]. Management of TD is different than the management of acute EPS. Anticholinergic drugs are not recommended (actually, these drugs have been shown to exacerbate TD). The primary step is, according to guidelines, switching from the causative agent to an SGA followed by, if necessary, additional pharmacological treatment. An empirical treatment algorithm from Margolese et al. suggests tapering of anticholinergic drugs, switching to an SGA and, if necessary addition of tetrabenazine. Finally adding experimental therapy including donepezil/melatonin/vitamin E/vitamin B6/branched-chain amino acids (BCAAs) should be considered if previous steps do not provide relief [43]. Clozapine is considered the safest, even beneficial, SGA regarding TD due its ability to improve involuntary symptoms [41]. A recent prospective cohort study on TD incidence amongst outpatients on antipsychotic maintenance therapy showed some disappointing results regarding SGAs and TD incidence. While most of the previously conducted studies showed that the risk of TD with SGAs is one-quarter that of FGAs, the results of this study suggest that the risk with SGAs is more than half that of FGAs (excluding clozapine patients) or more than two-thirds of the risk (including clozapine patients) [44]. The finding of surprisingly high rate of TD among clozapine patients in this study was attributed to certain confounding factors, such as confounding by indication (prescribing of clozapine to patients with TD or at-risk for TD), and should be interpreted with caution. In CATIE study, patients with TD were excluded from being randomized to perphenazine treatment. There were no statistically significant differences in the rate of new onset TD across the group of antipsychotic drugs. The rates ranged from 13% (quetiapine) to 17% (perphenazine) [13]. Since patients in the FGA (perphenazine) group were free from previous TD, CATIE study does not enable true comparison between FGAs and SGAs regarding TD, but it offers some valuable insight into predisposing factors for TD registered as baseline. These factors are older age, previous exposure to FGA and anticholinergic medication, previous longer antipsychotic treatment, and acute EPS [13, 24]. The CUtLASS-1 study showed unexpectedly the increase of TD incidence in the SGA group of patients during the 12th week of treatment, but this was probably due to switch of treatment (withdrawal of D2 blocking drug and the initiation of an SGA with more anticholinergic effects). This difference in the TD incidence was diminished by 52nd week of the followup [39].

Recent studies on the propensity of FGAs and SGAs to cause EPS yielded conflicting results [35, 37, 39, 45]. When interpreting these studies, it is of utmost importance to consider methodological issues and limitations, some of which are doses of antipsychotics, choice of an FGA comparator, duration of the study, inclusion and exclusion criteria, baseline patients' characteristics, and sensitivity of the criteria for EPS.

EPS remain the most serious problem among patients affected with schizophrenia, even in the era of new antipsychotics with less affinity towards D2 receptors. Upon the introduction of second-generation antipsychotics, these agents were defined as atypical based on their mechanism of action. Atypical antipsychotics expressed less affinity for striatal D2 receptors than typical, FGAs, and different levels of 5-HT2A antagonism, alpha-1 antagonism, or cholinergic antagonism. However, all SGAs still affect D2 receptors to some degree, with clozapine having the least affinity [7, 46] and therefore have some nonnegligible EPS liabilities.

## 3. Conclusion

SGAs have not completely fulfilled the expectation of being EPS-free antipsychotic drugs. Though recommended by current guidelines as the first-line therapy in the treatment of schizophrenia [47], the superiority of these drugs in terms of better efficacy and tolerability is not clear. Recent studies showed that SGAs do not significantly differ from FGAs in terms of efficacy (with the exception of clozapine for treatment-resistant patients) and have in general lower liability to cause EPS than FGAs, but with great variations within the class [48].

The likelihood of causing EPS with an SGA exists and depends on many factors. The patient's characteristics (age, gender, and concomitant conditions), history of the disease, previous treatment, the choice of a particular antipsychotic, its dose, and duration of treatment and adjuvant therapy should be taken into consideration in the order to minimize the risk of EPS and provide the best quality of care. At this moment, the trial-and-error approach is recommended, since the therapeutic outcome and adverse effects are not easily predictable. Hopefully, the recent, promising advances in pharmacogenomics and neurobiology could provide predictive markers of antipsychotic response and adverse effects and lead towards personalized therapy [48].

## Figures and Tables

**Table 1:** First- and second-generation antipsychotics and D2 antagonism.

Antagonistic D2 effect	First-generation antipsychotics	Second-generation antipsychotics
Low	Chlorpromazine Levomepromazine Thioridazine	Clozapine Quetiapine

Intermediate	Trifluoperazine Perphenazine	Olanzapine

High	Haloperidol Fluphenazine Flupentixol	Risperidone Ziprasidone Aripiprazole (possible D2 agonism)
